# Corn stover usage and farm profit for sustainable dairy farming in China

**DOI:** 10.5713/ajas.19.0222

**Published:** 2019-12-24

**Authors:** Yuan He, John W. Cone, Wouter H. Hendriks, Jan Dijkstra

**Affiliations:** 1 Animal Nutrition Group, Wageningen University & Research, De Elst 1, 6708 WD Wageningen, The Netherlands

**Keywords:** Profit, Dairy Farm, Corn Stover Silage, Whole Plant Corn Silage

## Abstract

**Objective:**

This study determined the optimal ratio of whole plant corn silage (WPCS) to corn stover (stems+leaves) silage (CSS) (WPCS:CSS) to reach the greatest profit of dairy farmers and evaluated its consequences with corn available for other purposes, enteric methane production and milk nitrogen efficiency (MNE) at varying milk production levels.

**Methods:**

An optimization model was developed. Chemical composition, rumen undegradable protein and metabolizable energy (ME) of WPCS and CSS from 4 cultivars were determined to provide data for the model.

**Results:**

At production levels of 0, 10, 20, and 30 kg milk/cow/d, the WPCS:CSS to maximize the profit of dairy farmers was 16:84, 22:78, 44:56, and 88:12, respectively, and the land area needed to grow corn plants was 4.5, 31.4, 33.4, and 30.3 ha, respectively. The amount of corn available (ton DM/ha/yr) for other purposes saved from this land area decreased with higher producing cows. However, compared with high producing cows (30 kg/d milk), more low producing cows (10 kg/d milk) and more land area to grow corn and soybeans was needed to produce the same total amount of milk. Extra land is available to grow corn for a higher milk production, leading to more corn available for other purposes. Increasing ME content of CSS decreased the land area needed, increased the profit of dairy farms and provided more corn available for other purposes. At the optimal WPCS:CSS, MNE and enteric methane production was greater, but methane production per kg milk was lower, for high producing cows.

**Conclusion:**

The WPCS:CSS to maximize the profit for dairy farms increases with decreased milk production levels. At a fixed total amount of milk being produced, high producing cows increase corn available for other purposes. At the optimal WPCS:CSS, methane emission intensity is smaller and MNE is greater for high producing cows.

## INTRODUCTION

In recent years, the profit of dairy farms in China decreased due to the lower price of raw milk and higher price of commonly used feedstuffs, such as ground corn (GC), soybean meal (SBM), and high quality forages [1]. Since feed costs are in general the greatest component of total operating expenses on most dairy farms, reducing feed costs would be an effective method to improve the profit of dairy farms. Utilization of crop residues may help dairy farmers to reduce feed costs [1].

The land area used to grow corn plants in China in 2014 has been estimated to be more than 3×10^7^ hectares [2], making corn an important economic crop for China. Whole plant corn silage (WPCS) is widely used in dairy farms globally. Corn stover silage (CSS), the silage of the stems and leaves obtained after harvesting the ears from the plants, is rarely included in a ration of high producing cows, due to its greater neutral detergent fiber (NDF) and acid detergent lignin (ADL) content and lower degradability, leading to a lower net energy (NE) content compared with WPCS [1]. Since low producing and dry cows require less energy than high producing cows, it may be possible to include CSS as a major forage source in the ration of low energy demanding cows without compromising their productive performance. More information on the proper ratio of the plants used for WPCS to those used for CSS (WPCS:CSS) will enable dairy farmers to make better decisions during the harvest of corn plants based on the production level at their farms. In addition, the chemical composition and ruminal degradability of WPCS and CSS differs among corn cultivars [3] and as such it is of interest to evaluate the influence of corn cultivars on the profit of dairy farms.

According to the Food and Agriculture Organization (FAO) of the United Nations, the global human population will increase to approximately 9.5 billion by 2050, and 70% to 100% more food is required compared to the current demand [4]. Due to the activities of rumen microbes, dairy cows can convert low quality forages, which is inedible for humans, into high quality human edible food (milk and meat). Corn grain is an important starch source in the ration of dairy cows, which is also a high quality food for humans. In light of the increased demand for food, more corn grain should be utilized as a food source. As such, increased utilization of CSS, instead of WPCS, in dairy rations would contribute to future food security. Currently it is unknown which proportion of maize plants may be harvested and used as CSS to maximize profit of dairy farms at different cow production levels, and consequently how much corn grain can be saved and be available for other purposes. Increasing food security, however, is likely to affect efficiency of production as replacing starch with fiber in dairy cattle diets may affect enteric methane (CH_4_) production [5] and milk nitrogen (N) efficiency (MNE) [6], two important parameters of sustainability.

The objectives of this study were to determine the optimal WPCS:CSS to maximize the profit of dairy farms, and to analyze how much corn grain can be used for other purposes, with varying WPCS:CSS and varying milk production levels, as well as to evaluate the consequences for enteric CH_4_ production and MNE. We hypothesized that the optimal WPCS: CSS ratio depends on milk production level, and that this in turn affects profit of dairy farms, corn available for other purposes, methane intensity and MNE to significant extents.

## MATERIALS AND METHODS

### Corn harvesting and ensiling

Corn plants from 4 different cultivars were collected and ensiled as WPCS and CSS and their nutritional value, including chemical composition, rumen undegradable protein (RUP) and metabolizable energy (ME), was determined, to provide data required for optimization of the model. Whole corn plants of 2 cultivars (Lg30248 and Perley) were harvested from a trial field in Wouw (the Netherlands) with a sandy soil of Limagrain (Rilland, the Netherlands) on September 2016, and another 2 cultivars (Rivaldinio and Leovoxx) were harvested from the experimental fields with a sandy soil of Unifarm in Wageningen (the Netherlands). All plants were cut at 10 cm above the ground. After harvesting, the ears of 5 plants per cultivar were separated from the leaves and stems (stover). Five whole plants as well as the five stovers were chopped into 1 to 2 cm pieces and ensiled in triplicate in 0.5 L clamp lid glass jars (IKEA, Leiden, the Netherlands) per cultivar. After weighing, approximately 300 g (wet weight) whole plant or 200 g (wet weight) corn stover was filled in each glass jar without a headspace. For Lg30248 and Perley, no molasses or lactic acid bacteria were added unlike Rivaldinio and Leovoxx. The application rate of molasses was 1% of the fresh forage weight [7], and 0.758 g *Lactobacillus plantarum* (*L. plantarum*) (Volac International Ltd., Royston, UK) was mixed with 2.5 L distilled water and 10 mL of the solution was applied to 1 kg fresh forage. The jars were stored at room temperature (~20°C) for 8 weeks. After ensiling, the samples were taken out of the glass jars and stored at −20°C in plastic bags.

The samples were thawed at room temperature and separated into two portions. Thirty g of each sample was weighed into a stomacher bag, diluted with 270 mL distilled water and mixed vigorously for 5 min, where after the fluid was used to determine pH and ammonia-N. The remainder was oven-dried at 70°C for 72 h and ground to pass a 1 mm sieve using a Peppink 100 AN cross-beater mill (Peppink, Deventer, The Netherlands) and stored in a plastic bottle until chemical analysis and *in vitro* gas production (IVGP).

### Chemical analysis

After the samples were mixed with distilled water as described above, the pH of the fluid was measured immediately using a pH meter (Hanna Instruments pH 300GLP, Amorim Povoa de Varzim, Portugal). Thirty mL of the fluid was centrifuged at 25,000×*g* for 10 min, and the supernatant (1.5 mL) was collected and acidified with equal volumes of trichloroacetic acid for ammonia analysis. Ammonia-N was determined by a colorimetric method, as described by Scheiner [8].

Dry matter (DM) content was determined gravimetrically after 4 h heating at 103°C in an oven and ash content after combustion for 3 h at 550°C in a muffle furnace. Ether extract content was determined using a Foss Soxtec 2050 (Foss, Hilleroed, Denmark), after extraction with petroleum ether. The NDF, acid detergent fiber (ADF) and ADL were determined by the methods as described by He et al [9,10]. Nitrogen was determined by the Kjeldahl method and crude protein (CP) was calculated as N×6.25. Nitrogen was determined in the NDF and ADF residues to calculate the neutral detergent insoluble nitrogen (NDIN) and acid detergent insoluble nitrogen (ADIN), as well as the CP in NDF (NDICP) and ADF (ADICP).

### *In vitro* gas production

The IVGP technique was performed to determine the amount of ME [11] and RUP [12] of WPCS and CSS. Rumen fluid was collected 2 h after the morning feeding from three non-lactating rumen fistulated cows fed a grass silage based diet twice daily. The rumen fluid was pooled, stored in warm insulated flasks, pre-filled with CO_2_ and filtered through 2 layers of cheesecloth. All experimental procedures with fistulated cows were conducted under the Dutch law (Experiments on Animals Act), in accordance with the European Directive 2010/63/EU.

For the ME prediction, the rumen fluid was mixed with an anaerobic buffer/mineral solution as described by Menke et al [11] under continuous flushing with CO_2_. A carefully weighed amount of DM (400 mg) of the ground samples was incubated in 60 mL buffered rumen fluid (one part of rumen fluid and two parts of buffer) in 250 mL bottles at 39°C in a shaking water bath. Each sample was run in one bottle each time and two runs were performed during separate weeks. Gas production was recorded for 24 h using an automated system [13].

For RUP prediction, 1 part of the rumen fluid was mixed with 19 parts of a N-free anaerobic buffer/mineral solution [12] under continuous flushing with CO_2_. Ten g/L rapidly fermentable carbohydrates (3.33 g/L glucose [Merck 8337, Merck, Darmstadt, Germany], 3.33 g/L xylose [X1500, Sigma-Aldrich, Darmstadt, Germany] and 3.33 g/L soluble starch [Merck 1252, Merck, Darmstadt, Germany]) were added to the buffered rumen fluid and incubated at 39°C for 4 h in a 5 L bottle with continuous flushing of CO_2_. After 4 h incubation, 60 mL of the buffered rumen fluid was added with a dispenser to bottles which contained exactly 15 mg N originating from the sample. Each sample was run in one bottle each time and two runs were performed during separate weeks. Gas production was recorded for 48 h with an automated system [13].

The equations used to predict the ME and RUP contents of the silage samples were described by Menke et al [11] and Cone et al [12], respectively.

### Model development

A reference dairy farm was defined with 100 dairy cows, 305 days lactation and 60 days dry period. The mature weight of the cows was assumed to be 650 kg [14]. The average CP, crude fat and lactose content in the milk was assumed to be 3.22% [15], 3.81% [15], and 4.85% [14], respectively. It was further assumed that the cows had no net gain or loss of body weight and were not pregnant. A simple ration, consisting of GC, SBM, and forages (WPCS and CSS) was formulated to calculate the feed costs using the income over feed costs principle [16] and to meet the NE and metabolizable protein (MP) requirements of the cows according to the NRC [14] whilst maximal voluntary DMI and diet NDF content restrictions also applied (described further on). The energy contents (digestible energy, ME and NE) of the feedstuffs which were used in the model, were derived from NRC [14] based on their chemical composition and total NE requirement of the cows. The chemical composition of GC and SBM were also obtained from the NRC [14], while the chemical composition of CSS and WPCS were determined as described above since the RUP of WPCS (with DM content being 39.2% and 39.7%) and CSS were not reported by the NRC [14]. The fermentation parameters and chemical composition of WPCS and CSS of the 4 corn cultivars are shown in Table 1 and the average values were used in the model. The difference in DM content of the 4 corn cultivars were mainly caused by the harvest date, with advanced maturity stage for Rivaldinio and Leovoxx explaining the higher DM content of these varieties. The digestibility of the RUP of WPCS and CSS was predicted by the equations described by Givens et al [17]. The yield of microbial CP (MCP) was calculated as 0.130×discounted total digestible nutrients (TDN) when rumen degradable protein (RDP) intake exceeded 1.18×TDN-predicted MCP; otherwise, the yield of MCP was calculated as 0.85×RDP intake [14]. The MCP was assumed to contain 80% true protein and the true protein was assumed to be 80% digestible [14]. Except RUP and MCP, endogenous CP (ECP) also contributed to MP and the conversion of ECP to MP was 40% based on the NRC [14]. To maintain a healthy rumen environment, the minimum amount of NDF included in the ration of the cows was set at 25% of the feed intake [14]. The maximum amount of NDF was set according to Mertens [18], with a maximum amount of NDF included in the ration of cows producing 0, 10, 20, and 30 kg milk daily of 60%, 53%, 45%, and 37%, respectively, beyond which the dry matter intake (DMI) is depressed. Maximal DMI was another constraint in optimization and was based on NRC [14] with milk production level as a major determinant of maximal DMI. Actual DMI as obtained in the optimization procedure (described further on) was always lower than the maximal DMI. Based on the NE and MP requirements of the dairy cows and the nutrients provided by the feedstuffs, together with the prices of the feedstuffs and the costs of make silage, the least cost ration can be formulated at different WPCS:CSS ratios.

In the present analysis, the profit of dairy farmers was defined as the difference between the income (selling raw milk) and the feed costs. The average price of raw milk was taken to be 3.45 renminbi (RMB)/kg [19]. The dairy farmers bought GC and SBM from the market and whole plant corn and corn stover from arable farmers, and then made WPCS and CSS at the farms. Since the objective of this model was to evaluate whether the profit of dairy farmers changed with decreasing WPCS:CSS, which had no influence on the salaries, electricity, other fixed costs, etc., only the costs related to the feedstuffs and silage making (machine, cover film, molasses and lactic acid bacteria) were considered. The price of GC and SBM was assumed to be 2.37 and 3.38 RMB/kg fresh weight, respectively [19]. The price of corn stover was assumed to be 50 RMB/ton forage (fresh weight), due to its low nutritional value. The price of corn grain sold by the arable farmers was arbitrarily set at 60% of the price of GC bought at the market. The price of whole plant corn sold by the arable farmers was calculated based on the profit of selling corn grain and corn stover and the ratio of corn stover to whole plant corn (assumed to be 0.41) and corn grain to corn stover (assumed to be 1.11) on DM basis [3] to make the profit of arable farmers (RMB/ha/yr) constant with different WPCS:CSS ratios. In this case, the price of whole corn plants was 325 RMB/ton forage (fresh weight). Mechanical processing is a commonly used procedure during whole corn plants harvesting, which can decrease the proportion of intact corn kernels present in WPCS and improve the total tract starch digestibility [20]. Therefore, when the plants were harvested and ensiled as WPCS, the costs for the machinery to harvest whole plants was assumed to be greater, and the machine used to harvest corn plants as WPCS and CSS was assumed to cost 1,500 and 1,000 RMB/hectare, respectively. The price of the cover film was set at 2.4 RMB/m2. For simplicity, the bunker silo was assumed to be cubic requiring three surfaces to be covered by the film. The surface of the silo was calculated based on the silo volume, which was related to the weight and density of the silage. The weight of the silages was calculated according to the daily intake, number of cows and days. The density of WPCS [21] and CSS [22] was 232 and 140 kg DM/m^3^, respectively. The application rate of molasses was 1% of fresh forage weight [7] and 0.758 g *L. plantarum* (Volac International Ltd., Royston, UK) was mixed with 2.5 L distilled water and applied to 250 kg forage. The price of molasses and *L. plantarum* was set at 900 RMB/ton and 2,500 RMB/kg, respectively.

In the present analysis, the profit of arable farmers was defined as the difference between their income (selling whole plant corn and/or corn stover and corn grain separately when the plants were harvested as CSS, all mentioned above) and the costs to grow the corn plants and to collect the corn grain from the ear. According to the National Development and Reform Commission [23], the average costs, including labor, land, fertilizer etc. to grow corn plants is 12,415 RMB/ha. After harvest, the arable farmers could sell the corn grain to feed or food companies to be further processed such as drying and grinding while the dairy farmer receives the corn stover. It was assumed that the cost to separate the corn grain from one ear by a machine is 0.01 RMB.

The weight of the corn stover of one plant was taken to be 0.0859 kg on DM basis [10]. Due to the fluid leakage and microbial fermentation during ensiling, the DM loss was reported to be 10% for WPCS [24] and it was assumed that this was also applicable for the CSS.

For each cow production level, the non-linear Generalized Reduced Gradient of the Solver program in Excel was used to calculate diet composition to obtain the maximum profit of the dairy farmer whilst adhering to nutritional constraints. The total amount of WPCS and CSS required was calculated based on the daily intake of one dairy cow, the number of total cows at the farm and lactating days. The number of plants needed for this amount of WPCS and CSS was derived from the data (DM loss during ensiling and DM production of whole plant corn and corn stover) as described above. The total weight of corn grain, which was produced from the plants needed to make the CSS, was calculated according to the ratio of the weight of corn grain to corn stover on DM basis. The corn available for other purposes referred to the corn grain which was potentially available for human consumption or animal feed, and was assumed to be the difference between the corn grain harvested when the plants were used as CSS and corn grain (GC) purchased from the market.

The sowing density of corn plants was 10/m^2^, with 13.3 cm between plants and 75 cm between the rows [9], which was used to calculate the total land area required to grow corn plants. The soybean yield was 2.76 ton/ha [25] and 78.7% of the soybean (fresh weight basis) can be SBM [26] with a DM content being 89.5% [14]. To investigate the effect of the different milk production levels on the total corn available for other purposes comprehensively, an annual milk production was set at 915 ton (being the total amount of milk of 1 farm with 100 cows producing 30 kg/d for 305 days) to calculate the number of cows needed, land area needed to grow corn and soybean for the dairy farms, extra land used to grow corn, and total corn available for other purposes. Land area to grow corn plants and soybean was calculated based on the ration to reach the highest profit of dairy farms, the number of cows, the lactating days and the yield of whole plant corn, corn stover and SBM. Corn grain available for other purposes, originating from the land area to grow corn plants for the dairy farms, was calculated as the amount of corn grain harvested when the corn plants are used as CSS, minus corn grain purchased from the market. The total land area needed to grow corn plants and soybean for the three milk production levels of cows to achieve the annual milk production of 915 ton was calculated. Extra land area relative to the total land area required at a 10 kg/d milk production level was calculated, and this extra land area was assumed to be used to grow corn plants, which will become available for other purposes. The total corn available for other purposes was the sum of the corn grain saved from the land needed to grow corn plants for dairy farms and corn grown on the extra land.

The enteric CH_4_ production was calculated based on the diet composition and the assumption that the enteric CH_4_ value of WPCS, CSS, GC, and SBM was 17.5, 17.0, 19.7, and 20.5 g CH_4_/kg DM, respectively [27,28]. Milk N efficiency was calculated as N in milk divided by N intake.

### Sensitivity analysis

The sensitivity of the model to changes in parameters related to the nutritional value of the forages and the price of the feedstuffs was investigated. Two corn cultivars with the highest and lowest predicted ME content (using NRC [14] equations) of CSS (Perley and Rivaldinio, respectively) were selected to test the model sensitivity. Since the price of GC corn varies during the year, the highest and lowest price (year 2015) were used in the sensitivity analysis. The price of corn stover was set at ±20% of the model value to evaluate sensitivity.

## RESULTS

The diet composition for dry cows and for lactating cows producing 10, 20, and 30 kg milk per day during 305 lactating days, when 0%, 25%, 50%, 75%, and 100% of the total number of corn plants were used to produce CSS (equivalent to 100%, 75%, 50%, 25%, and 0% of the total number of corn plants used to produce WPCS, respectively) is shown in Table 2. Within a milk production level, upon increasing the CSS proportion of the diet, the DMI initially increased and then decreased. Cows producing more milk had a greater DMI and greater proportion of GC and SBM in the diet, related to their greater nutritional demands. When 100% of the plants were used to produce CSS, GC was included in the diets for all the cows independent of milk production level.

Corn plants and land area required, annual profit of both dairy farmers and arable farmers and corn available for other purposes, for 100 dry cows during the 60 days dry period and 100 lactating cows at different production levels during 305 lactating days when 0%, 25%, 50%, 75%, and 100% of plants are used as CSS, are shown in Table 3. More plants were needed when more CSS was included in the diets, given the lower nutritional value of CSS compared with WPCS. The profit of dairy farmers initially increased and then (at the point where GC was included in the diet) decreased with the increased WPCS:CSS ratio. To reach the greatest profit of dairy farmers, the optimal WPCS:CSS ratio increased with greater milk production levels, with optimal WPCS:CSS ratios ranging from 16:84 for dry cows to 88:12 for cattle producing 30 kg milk daily. The annual profit of arable farmers increased with decreasing WPCS:CSS. More corn (ton DM/ha/yr) is available for other purposes if more plants were used as CSS in the diets of cows. However, at similar WPCS:CSS ratios, elevated milk production levels decreased the amount of corn grain available for other purposes, because at elevated milk production levels a greater proportion of the diet consists of corn grain.

The number of cows needed to achieve 915 ton/yr milk production (being the total amount of milk of 1 farm with 100 cows producing 30 kg/d for 305 d), land area needed to grow corn and soybean for the dairy farms under different milk production levels, and total corn available for other purposes, are shown in Table 4. To achieve the same milk production with 100 cows producing 30 kg milk daily for 305 days, 150 and 300 cows are required at production levels of 20 and 10 kg/d, respectively. Corn available for purposes other than to be included in the diet for dairy cattle, and only from the land directly needed to grow corn plants to make silage (the sum of WPCS and CSS), decreased with the increased milk production level. To achieve the same amount of milk production, more land area was needed to grow corn plants to make silage and soybean for feed with cows producing less milk daily. To produce 915 ton milk/yr at a production level of 10 kg/d, 149.9 ha is required, whereas at production levels of 20 kg/d and 30 kg/d, 100.6 and 77.2 ha is required, respectively. Therefore, compared with a milk production level of 10 kg/d, at milk production levels of 20 and 30 kg/d, 49.3 and 72.7 ha land was available for other purposes, respectively. Assuming that this 49.3 and 72.7 ha land was also used to grow corn plants for corn grain, the total corn available for other purposes was highest at a milk production of 30 kg/d, closely followed by a milk production of 20 kg/d, and with the lowest amount of corn available for other purposes at production levels of 10 kg/d.

The enteric CH_4_ production and MNE of cows producing 10, 20, and 30 kg milk daily are shown in Table 5. The CH_4_ production (g/d), CH4 intensity (g/kg milk) and N intake of cows were highest, and the MNE was lowest, when the WPCS: CSS was optimal to reach the highest profit of the dairy farms at each milk production level. Upon an increase in milk production level from 10 to 30 kg/d, daily CH_4_ production of cows increased (from 205 to 365 g/d) while CH_4_ intensity decreased (from 20.5 to 12.2 g/kg). The cows with higher milk production had a higher MNE (33.7% and 25.4% at 30 and 10 kg/d, respectively).

The effect of the quality and price of CSS and the price of GC on the land area required to grow corn plants, the profit of dairy farmers and arable farmers and corn saved are shown in Supplementary Table S1. Two corn cultivars, Perley and Rivaldinio, were selected to do the sensitivity analysis. The CSS of Perley and Rivaldinio had the greatest and lowest predicted ME by the NRC [14], respectively. The quality of CSS influenced the land area required to grow corn plants and the profit of the dairy farmers. Compared with the cultivar Perley, when Rivaldinio was the corn cultivar selected, more land area was needed to grow the plants (up to 3.0 ha more), except when all the plants were used as WPCS for dry cows and as CSS in the farms with cows producing 30 kg milk daily (in both situations, land area required not different for both cultivars). Rivaldinio, in contrast to Perley, increased the profit of dairy farms with cows producing 20 and 30 kg/d milk when WPCS:CSS was 100:0. The corn cultivars (Rivaldinio and Perley) selected did not affect the profit of dairy farms with dry cows when WPCS:CSS was 75:25. In most cases though, Perley was a better corn cultivar than Rivaldinio in terms of dairy farm profit, with a maximum difference in profit being 0.044 million RMB/ha. The CSS quality affected the corn available for other purposes when all the plants were harvested as CSS to feed dry cows and cows with 10 kg milk production daily, more than 75% of the plants as CSS to feed cows with 20 kg milk production daily and more than 25% of the plants as CSS to feed cows with 30 kg milk production daily. Perley saved more corn for other purposes than Rivaldinio in all the cases described above, with a maximum of 0.8 ton DM/ha extra for Perley compared with Rivaldinio. No effect of the quality of CSS on the price of WPCS and the profit of arable farmers was observed. In contrast, the price of CSS and GC had no influence on the land area needed and corn available for other purposes. The lower price of CSS and GC led to a lower price of WPCS, a lower profit of the arable farmers and a greater profit of the dairy farmers, where profit was up to 0.019 million RMB/yr higher at lower CSS price, and up to 0.093 million RMB/yr at lower GC price. A greater price of CSS and GC resulted in a greater price of WPCS, a greater profit of the arable farmers and a lower profit of the dairy farmers.

## DISCUSSION

The main objective of this study was to evaluate the impact of a change of the WPCS:CSS ratio on the profit of dairy farmers, thus providing guidance for an effective utilization of corn plants. Since the arable farmers also aim to obtain maximum profit, it was necessary to keep the profit of the arable farmers constant with varying WPCS:CSS ratios. In the default situation, the profit of arable farmers was 3,281 RMB/ha/yr. The price of corn stover is always relatively low due to its high lignin content and low degradability [1]. In our model, the price of fresh corn stover sold by arable farmers was 50 RMB/ton. According to Shi [29], the price of WPCS can be set based on the weight of one corn plant, corn grain and corn stover and the price of corn grain and corn stover to make the profit of arable farmers be constant, regardless of the WPCS:CSS ratio. As such, the profit of the arable farmer is influenced by the price of corn stover and GC, but not by the quality of the CSS.

### Effect of the WPCS:CSS ratio on diet composition and profit of dairy farms

The GC, SBM, WPCS, and CSS are commonly used as major feedstuffs in the ration of dry and lactating cows in Chinese dairy farms [30]. In our model, a simple ration consisting of GC, SBM, WPCS, and/or CSS [16] was formulated to meet the requirements for NE and MP of cows at different milk production levels.

With no GC included in the ration, the DMI increased with more CSS included to meet the NE requirement of cows, due to the lower energy content of CSS compared with WPCS. This is consistent with Mertens [18] who suggested that DMI is positively correlated with NDF concentration when energy limits the intake. Besides, DMI is negatively correlated with NDF concentration when fill of the reticulo-rumen limits intake [18]. Hence, in our model, the maximum amount of NDF included in the ration decreased with increasing milk production level and thus increasing DMI level. When the NDF content in the ration reached this maximum, GC was selected over more CSS in the ration. The greater energy density of GC compared to CSS, contributes to the lower DMI when more GC is added.

Endogenous N losses increase with elevated DMI levels [14], which results in a greater amount of MP required when feed intake increases. The lower MP content in CSS compared with WPCS also resulted in an increasing demand of MP from GC and SBM. Hence, the fraction of SBM in the ration increased with lower WPCS:CSS ratios. However, the greater MP content in GC, compared with CSS, can alleviate the usage of SBM. Because the price of CSS was lower than WPCS and the price of corn grain sold by the arable farmers was lower than GC bought by the dairy farmers in the market (as is the case in China), the profit of dairy farmers increased with lower WPCS:CSS ratios, until GC was included in the ration where the WPCS:CSS ratio was the highest.

### Effect of the CSS quality on diet composition and profit of dairy farms

**Figure 1:**
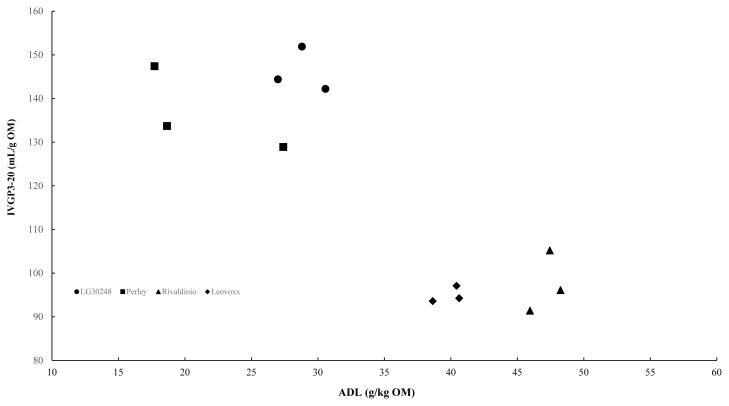
Relationship between acid detergent lignin (ADL) content (g/kg organic matter, OM) and *in vitro* gas production (IVGP) between 3 and 20 h of incubation in buffered rumen fluid (IVGP3–20; mL/g OM) of corn stover silage from four cultivars (Lg30248, Perley, Rivaldinio and Leovoxx): IVGP3–20 = −1.94±0.386×ADL+185±13.7 (estimate±standard error), root-mean-square-error.

The NDF degradability is of vital importance for dairy cows in terms of milk production and DMI. Oba and Allen [31] proposed that a 1-unit increase in forage NDF degradability *in vitro* or *in situ* is associated with an increase of 0.17 kg DMI/d and 0.25 kg 4.0% fat-corrected milk/d. In previous studies, the lignin content in forages was shown to be negatively correlated to cell wall degradability [9]. One commonly used method to evaluate the cell wall degradability is the IVGP technique. The IVGP between 3 and 20 hours (IVGP3–20) is assumed to represent the cell wall degradability of forages [9]. The relationship between ADL content expressed on an organic matter basis and IVGP3–20 of CSS is shown in Figure 1. The ADL content of CSS has a good correlation (adjusted R^2^ = 0.69) with the cell wall degradability of CSS. A greater NDF degradability will result in a greater ME content, and CSS of Perley (lowest ADL content) indeed had the greatest ME content, while Rivaldinio (highest ADL content) had the lowest ME content. Considering both the predicted ME value from NRC [14] and the NDF degradability evaluated using the IVGP of CSS from the 4 cultivars, Perley and Rivaldinio were selected for the sensitivity analysis.

Since the aim of our model was to formulate rations for cows with a pre-defined milk production, the beneficial effects of a greater NDF degradability on milk production or DMI was not observed. However, due to the greater ME value of CSS from Perley relative to Rivaldinio, less feed is required to meet the energy demand of the cows (Table 6), indicating that feed efficiency of cows fed with Perley corn plants was higher than that of cows fed with Rivaldinio, and more corn grain can be saved for other purposes (Table 6). The greater ME value of Perley than Rivaldinio is one of the factors that can help to reduce the land area needed to grow the plants, which can also be influenced by the NDF content in Perley and Rivaldinio and in the ration. When the NDF content in the ration did not reach the limit, which was set in our model based on the production levels, the inclusion rate of Perley in the ration was lower than that of Rivaldinio. When the NDF content in the diet limited the inclusion of CSS, more Perley could be incorporated due to its lower NDF content. However, Perley contained less CP than the other three corn cultivars, which resulted in the inclusion of more SBM. Therefore, the use of Perley did not benefit the profit margin of dairy farmers in some cases. Collectively, the results indicate that corn plants with a greater NDF degradability may not only be beneficial for cows in early lactation, during which energy intake cannot meet the requirements due to the low DMI, but also contribute to more corn available for other purposes.

### Effect of milk production levels on land use, CH_4_ emission and MNE

Due to the low NE requirement, the lower producing dairy cows require less corn grain than the high producing cows, and at a fixed number of cows, more corn grain is available for other purposes from the land needed to grow corn for dairy farms with low producing cows (Table 4). However, the amount of corn saved for other purposes should be placed into the perspective of the amount of milk produced. Therefore, a fixed level of 915 ton/yr milk production, which was the milk production from one dairy farm with 100 dairy cows producing 30 kg/d milk and having 305 lactating days and 60 d dry period, was set as a goal to be achieved by dairy farms with cows producing 10 and 20 kg/d milk. At this fixed total milk production level, more land area is required upon a reduction in milk production level per cow, and more land is available to grow corn at high milk production level per cow. Therefore, our simulations indicate that in total a greater amount of corn will be available for other purposes when dairy cows produce 20 and 30 kg milk/d, compared with cows producing 10 kg milk/d. All cows, regardless of milk production levels, are assumed to have the same NE requirement for maintenance. Therefore, upon an increase in milk production level, a smaller proportion of feed energy intake is partitioned toward meeting maintenance needs and a greater proportion is transferred to milk. The 300 cows with 10 kg/d milk production need more NE to produce 915 ton/yr milk than the 150 cows with 20 kg/d milk production and the 100 cows with 30 kg/d milk production, resulting in the less corn saved for other purposes. A discount factor is applied to predict the digestible energy of feedstuffs at different production levels, and the factor is larger when cows have a greater milk production [14]. Although the cows producing 30 kg milk/d are more efficient than the cows producing 20 kg milk/d in terms of energy, the supply of digestible energy per unit feed is lower, resulting in only a small increase in corn available for other purposes when milk production increases from 20 to 30 kg/d.

Methane emission from ruminants receives global interest. Van Gastelen et al [5] found that replacing grass silage with WPCS for dairy cattle is an effective strategy to decrease enteric CH_4_ production without negatively affecting dairy cow performance. In the present study, within a certain milk production level, the increased CH_4_ production is mainly caused by replacing starch-rich WPCS with fiber-rich CSS. Fermentation of fiber favors the ruminal production of acetic acid, which increases hydrogen availability and activity of rumen methanogens [32]. We assumed a fixed yield of CH_4_ per kg DM of WPCS; increasing harvest maturity of WPCS may reduce CH_4_ yield [33] and may offer opportunities to further reduce CH_4_ emissions from dairy cattle. Not only the dietary composition (particularly the type of carbohydrates), but also the level of feed intake influences the enteric CH_4_ production [34]. It is without doubt that the high producing cows emit greater amounts of CH4 since they have a higher level of DMI. However, the amount of CH_4_ produced should be placed in the context of the amount of milk produced. It can be seen from our simulations that high producing cows have lower CH_4_ intensity. Nitrogen emission to the environment, which is related with the N efficiency, is another environmental concern in dairy production. The results in the present study demonstrated that greater milk production has the potential to improve MNE, which is similar to the finding of Dijkstra et al [35] that the maximum MNE increases upon an increase in milk production from 3,000 to 9,000 kg/yr. Nadeau et al [36] reported that MNE decreased with decreasing milk yield, with 32.8% for cows producing more than 35 kg milk/d, 30.6% for cows producing 25 to 35 kg milk/d and 26.8% for cows producing less than 25 kg milk/d. The MNE of cows producing 20 and 30 kg milk/d that was reported by Nadeau et al [36] was lower than the results in our study, mainly because the cows in the present model were assumed to be non-pregnant and there was no MP partitioned to the demand of pregnancy. The increased MNE of cows producing greater amounts of milk may be the result of a greater proportion of MP being partitioned to milk with greater amount of MP intake since the cows in different production levels are assumed to have the same MP requirement for maintenance.

## CONCLUSION

The optimal WPCS:CSS ratio to reach the greatest profit of dairy farmers changed with the production level of the cows, with a lower WPCS:CSS ratio for cows with a lower milk production. At a fixed total amount of milk being produced, more land will be available to grow corn and more corn will be available for other purposes including human consumption, with high producing cows. At the optimal WPCS:CSS ratio for each milk production level, CH_4_ emission intensity is smaller, and MNE is greater, with high producing cows compared with low producing cows, which may alleviate the environment pollution related to dairy production.

## Figures and Tables

**Table 1:** Chemical composition (g/kg DM, unless stated otherwise) and fermentation parameters of whole plant and stover (stems+leaves) silage of 4 corn cultivars

Silage	Cultivar	DM (g/kg)	pH	NH_3_-N (% of N)	Ash	CP	EE	NDF	ADF	ADL	NDICP	ADICP	RUP1) (g/kg CP)	ME2) (MJ/kg DM)		

														a	b	c
WPCS	Lg30248	397	4.70	6.94	34	71	30	368	206	9	8	3	420	12.26	10.96	9.92
	Perley	392	4.68	4.96	30	62	33	390	217	12	9	4	418	12.18	10.84	9.62
	Rivaldinio	501	3.80	1.81	35	70	31	390	224	20	11	4	305	11.76	10.59	9.46
	Leovoxx	448	3.89	2.70	36	78	25	455	232	17	14	3	358	11.51	10.46	9.58
CSS	Lg30248	271	4.63	8.71	66	48	16	683	413	27	11	4	351	9.12	8.87	7.36
	Perley	247	4.71	9.38	53	38	17	699	411	20	10	3	439	9.46	9.08	7.41
	Rivaldinio	449	3.81	3.52	56	49	10	719	458	45	13	6	420	8.41	8.41	5.69
	Leovoxx	349	4.10	10.69	63	63	11	708	422	37	15	5	425	8.70	8.66	5.52
	WPCS average	434	4.27	4.10	34	70	30	401	220	15	10	4	375	11.92	10.71	9.62
	CSS average	329	4.31	8.08	60	49	13	702	426	32	12	5	409	8.91	8.74	6.49

DM, dry matter; NH_3_-N, ammonia-nitrogen; CP, crude protein; EE, ether extract; NDF, neutral detergent fiber; ADF, acid detergent fiber; ADL, acid detergent lignin; NDICP, neutral detergent insoluble crude protein; ADICP, acid detergent insoluble crude protein; RUP, rumen undegradable protein; ME, metabolizable energy; WPCS, whole plant corn silage; CSS, corn stover silage.

1) RUP is predicted by *in vitro* gas production based on Cone et al [12].

2) ME values calculated by NRC [14] equations at (a) maintenance level, (b) 3×maintenance level and by (c) *in vitro* gas production based on Menke et al [11].

**Table 2:** Diet composition for dry cows and lactating cows (10, 20, and 30 kg/d) when fed various ratios of whole plant corn silage and corn stover silage

Milk yield (kg/d)	WPCS:CSS	DMI (kg/d)	Diet composition1) (%)			

			WPCS	CSS	GC	SBM
02)	100:0	6.3	100.0	0	0	0
	75:25	6.5	88.0	12.0	0	0
	50:50	6.7	70.8	29.0	0	0.2
	25:75	7.1	44.4	54.6	0	1.0
	0:100	7.1	0	83.2	15.5	1.3
103)	100:0	10.6	90.8	0	0	9.2
	75:25	10.8	79.7	10.9	0	9.4
	50:50	11.1	64.1	26.3	0	9.6
	25:75	11.6	40.3	49.6	0	10.1
	0:100	11.4	0	71.6	18.3	10.1
204)	100:0	15.2	87.0	0	0	13.0
	75:25	15.5	76.4	10.4	0	13.2
	50:50	15.9	61.4	25.2	0	13.4
	25:75	15.7	33.7	41.4	11.4	13.5
	0:100	15.4	0	58.4	28.1	13.5
305)	100:0	20.3	85.4	0	0	14.6
	75:25	20.3	70.6	9.6	5.1	14.7
	50:50	20.1	49.5	20.3	15.5	14.7
	25:75	19.8	26.1	32.1	27.1	14.7
	0:100	19.6	0	45.2	40.1	14.7

WPCS, whole plant corn silage; CSS, corn stover silage; DMI, dry matter intake; GC, ground corn; SBM, soybean meal.

1) The price of whole corn plant, corn stover, GC and SBM was assumed to be 0.87, 0.16, 2.69, and 3.78 RMB/kg DM, respectively.

2) The optimal WPCS:CSS ratio is 16:84 to reach the greatest DMI (7.3 kg/d).

3) The optimal WPCS:CSS ratio is 22:78 to reach the greatest DMI (11.7 kg/d).

4) The optimal WPCS:CSS ratio is 44:56 to reach the greatest DMI (16.0 kg/d).

5) The optimal WPCS:CSS ratio is 88:12 to reach the greatest DMI (20.4 kg/d).

**Table 3:** Corn plants and land area needed, annual profit of both dairy farmers and arable farmers, profit of arable farmers per hectare land, and corn saved, for dry cows during 60 days and lactating cows (10, 20, and 30 kg/d) during 305 days on a dairy farm with 100 cows when fed various ratios of whole plant corn silage and corn stover silage

Milk yield (kg/d)	WPCS:CSS	Plants needed (million)	Land area required (ha)	Profit dairy farmers (million RMB/yr)	Profit arable farmers (thousand RMB/yr)	Corn available for other purposes1)	

						ton DM/yr	ton DM/ha/yr
02)	100:0	0.20	2.0	−0.036	6.6	0	0
	75:25	0.24	2.4	−0.035	7.9	5.8	2.4
	50:50	0.30	3.0	−0.032	9.9	14.4	4.8
	25:75	0.40	4.0	−0.028	13.1	28.6	7.2
	0:100	0.46	4.6	−0.033	15.0	37.0	8.1
103)	100:0	1.56	15.6	0.658	51.2	0	0
	75:25	1.86	18.6	0.670	61.0	44.4	2.4
	50:50	2.30	23.1	0.688	75.6	109.9	4.8
	25:75	3.03	30.3	0.718	99.3	216.6	7.2
	0:100	3.21	32.1	0.661	105.3	242.7	7.6
204)	100:0	2.15	21.5	1.489	70.4	0	0
	75:25	2.55	25.5	1.507	83.7	60.8	2.4
	50:50	3.15	31.5	1.532	103.4	150.2	4.8
	25:75	3.43	34.3	1.487	112.5	190.5	5.6
	0:100	3.55	35.5	1.411	116.5	206.6	5.8
305)	100:0	2.80	28.0	2.311	91.8	0	0
	75:25	3.09	30.9	2.290	101.3	42.2	1.4
	50:50	3.21	32.1	2.227	105.3	58.0	1.8
	25:75	3.35	33.5	2.159	109.7	75.1	2.2
	0:100	3.49	34.9	2.086	114.5	93.6	2.7

WPCS, whole plant corn silage; CSS, corn stover silage; DM, dry matter; RMB, renminbi.

1) Corn grain potentially available for other purposes, calculated as the difference between corn grain harvested when the plants are used as CSS and corn grain purchased from the market.

2) The optimal WPCS:CSS ratio is 16:84 to reach the greatest profit of dairy farms (−0.026 million RMB/yr).

3) The optimal WPCS:CSS ratio is 22:78 to reach the greatest profit of dairy farms (0.722 million RMB/yr).

4) The optimal WPCS:CSS ratio is 44:56 to reach the greatest profit of dairy farms (1.541 million RMB/yr).

5) The optimal WPCS:CSS ratio is 88:12 to reach the greatest profit of dairy farms (2.321 million RMB/yr).

**Table 4:** Number of cows needed to achieve a milk production of 915 ton/yr, land area needed to grow corn plants and soybean, and total corn available for other purposes of cows producing 10, 20, and 30 kg milk daily at the greatest profit for dairy farms

Milk production (kg/d/cow)	10	20	30
Cows needed to achieve 915 ton milk production per year1)	300	150	100
WPCS:CSS to reach the highest profit of dairy farms2)	22:78	44:56	88:12
Land area needed to grow corn plants for dairy farms (ha)3)	94.2	50.2	30.3
Corn available for other purposes (ton/ha/yr, DM basis)4)	7.4	5.4	1.1
Corn grain saved from the land needed to grow corn plants for dairy farms (ton, DM basis)5)	699.2	268.3	34.5
Soybean meal needed for dairy farms (ton, DM basis)6)	108.3	98.1	91.3
Land area needed to grow soybeans for dairy farms (ha)7)	55.7	50.5	47.0
Land area needed to grow corn and soybeans for dairy farms (ha)	149.9	100.6	77.2
Extra land area to grown corn plants for corn grain (ha)8)	0	49.3	72.7
Corn grain harvested on the extra land area (ton, DM basis)	0	493.0	727.0
Total corn available for other purposes (ton, DM basis)	699.2	761.3	761.5

WPCS, whole plant corn silage; CSS, corn stover silage; DM, dry matter.

1) Assume that the lactating period is 305 d and dry period is 60 d.

2) Values were obtained from the model.

3) Values were calculated based on land areas to grow corn plants for one farm and the farm numbers.

4) Corn grain potentially available for other purposes, calculated as the difference between corn grain harvested when the plants are used as CSS and corn grain purchased from the market.

5) Values were the product of corn saved per ha and land area to grow corn plants.

6) Values were the product of the daily intake of soybean per cow, total number of cows and lactating days.

7) Soybean yield is 2.76 ton/ha [25], 78.7% (fresh weight basis) of the soybean is soybean meal [26], and DM of soybean meal is 89.5% [14].

8) Compared to the situation of cows producing 10 kg/d.

**Table 5:** Enteric methane production, nitrogen (N) intake and milk N efficiency of cows producing 10, 20, and 30 kg milk daily

Milk yield (kg/d)	WPCS:CSS	Methane production (g/d)	Methane intensity (g/kg milk)	N intake (g/d)	N efficiency1) (%)
10	100:0	189	18.9	192	26.3
	75:25	192	19.2	193	26.1
	50:50	196	19.6	195	25.9
	25:75	204	20.4	198	25.5
	0:100	203	20.3	194	26.0
	22:78	205	20.5	199	25.4
20	100:0	273	13.6	319	31.6
	75:25	276	13.8	321	31.5
	50:50	282	14.1	323	31.3
	25:75	282	14.1	320	31.6
	0:100	281	14.0	315	32.1
	44:56	284	14.2	324	31.2
30	100:0	364	12.1	449	33.7
	75:25	365	12.2	448	33.8
	50:50	365	12.2	444	34.1
	25:75	364	12.1	439	34.5
	0:100	364	12.1	435	34.8
	88:12	365	12.2	449	33.7

WPCS, whole plant corn silage; CSS, corn stover silage.

1) Calculated as milk N yield (g/d) divided by N intake (g/d) ×100.

**Table 6:** Diet composition for dry cows and lactating cows (10, 20, and 30 kg/d) when fed various ratios of whole plant corn silage and corn stover silage of the corn cultivars Rivaldinio and Perley

Milk yield (kg/d)	WPCS:CSS	DMI (kg/d)		Diet composition (%)							

				WPCS		CSS		GC		SBM	
				
		Rivaldinio	Perley	Rivaldinio	Perley	Rivaldinio	Perley	Rivaldinio	Perley	Rivaldinio	Perley
0	100:0	6.4	6.2	100.0	99.2	0	0	0	0	0	0.8
	75:25	6.6	6.3	88.0	86.9	12.0	11.9	0	0	0	1.2
	50:50	6.9	6.4	70.6	69.6	29.0	28.5	0	0	0.4	1.9
	25:75	7.4	6.7	44.3	43.6	54.5	53.6	0	0	1.2	2.8
	0:100	7.4	6.7	0	0	80.9	83.6	18.1	13.3	1.0	3.1
10	100:0	10.7	10.4	90.6	89.2	0	0	0	0	9.4	10.8
	75:25	11.0	10.5	79.6	78.2	10.9	10.7	0	0	9.5	11.1
	50:50	11.4	10.7	64.1	62.8	26.3	25.7	0	0	9.6	11.5
	25:75	12.0	11.0	40.4	39.4	49.7	48.5	0	0	9.9	12.1
	0:100	11.7	10.9	0	0	69.7	72.0	20.7	15.9	9.6	12.1
20	100:0	15.4	14.9	86.8	85.4	0	0	0	0	13.2	14.6
	75:25	15.7	15.1	76.3	74.9	10.4	10.3	0	0	13.3	14.8
	50:50	16.1	15.4	61.5	60.1	25.2	24.7	0	0	13.3	15.2
	25:75	16.0	15.3	33.4	34.2	41.1	42.0	12.3	8.5	13.2	15.3
	0:100	15.7	15.0	0	0	56.8	58.7	30.1	26.1	13.1	15.2
30	100:0	20.4	19.9	85.2	83.9	0	0	0	0	14.8	16.1
	75:25	20.6	20.1	72.2	72.7	9.9	9.9	3.1	1.0	14.8	16.4
	50:50	20.3	19.8	49.8	50.6	20.5	20.8	15.0	12.4	14.7	16.2
	25:75	20.1	19.5	25.8	26.5	31.8	32.5	27.8	24.9	14.6	16.1
	0:100	19.8	19.2	0	0	44.0	45.4	41.6	38.6	14.4	16.0

WPCS, whole plant corn silage; CSS, corn stover silage; DMI, dry matter intake; GC, ground corn; SBM, soybean meal.
