# Supplementary material for: Corn stover usage and farm profit for sustainable dairy farming in China
Source: Anim Biosci. 2019 Dec 24;34(1):36–47. doi: 10.5713/ajas.19.0222 (PMC7888508; doi:10.5713/ajas.19.0222)
Supplement: Supplementary file 1 [file ajas-19-0222-suppl.pdf]

Supplementary Table S1. Sensitivity of land area needed, profit of dairy farmer and arable farmer, and corn available for other purposes, to changes in quality of corn stover silage (CSS) and the price of CSS and ground corn (GC).

Milk yield, kg/d			Default[a]	CSS quality[b]		CSS price		GC price[c]	
				Rivaldinio	Perley	-20%	+20%	2.13	2.47
	Price of WPCS, RMB/kg		0.325	0.325	0.325	0.320	0.331	0.293	0.339
0	WPCS:CSS	Land area needed, ha	2.0	2.0	2.0	2.0	2.0	2.0	2.0
	100:0	Profit dairy farmer, million RMB/yr	-0.036	-0.037	-0.036	-0.036	-0.037	-0.033	-0.038
		Profit arable farmer, RMB/ha/yr	3281	3281	3281	3019	3542	1722	3930
		Corn saved, Mg DM/ha	0	0	0	0	0	0	0
	WPCS:CSS	Land area needed, ha	2.4	2.5	2.3	2.4	2.4	2.4	2.4
	75:25	Profit dairy farmer, million RMB/yr	-0.035	-0.035	-0.035	-0.034	-0.035	-0.032	-0.036
		Profit arable farmer, RMB/ha/yr	3281	3281	3281	3019	3542	1722	3930
		Corn saved, Mg DM/ha	2.4	2.4	2.4	2.4	2.4	2.4	2.4
	WPCS:CSS5	Land area needed, ha	3.0	3.1	2.8	3.0	3.0	3.0	3.0
	0:50	Profit dairy farmer, million RMB/yr	-0.032	-0.033	-0.032	-0.031	-0.033	-0.030	-0.033
		Profit arable farmer, RMB/ha/yr	3281	3281	3281	3019	3542	1722	3930
		Corn saved, Mg DM/ha	4.8	4.8	4.8	4.8	4.8	4.8	4.8
	WPCS:CSS2	Land area needed, ha	4.0	4.2	3.7	4.0	4.0	4.0	4.0
	5:75	Profit dairy farmer, million RMB/yr	-0.028	-0.030	-0.029	-0.027	-0.029	-0.027	-0.029
		Profit arable farmer, RMB/ha/yr	3281	3281	3281	3019	3542	1722	3930
		Corn saved, Mg DM/ha	7.2	7.2	7.2	7.2	7.2	7.2	7.2
	WPCS:CSS	Land area needed, ha	4.6	4.6	4.3	4.6	4.6	4.6	4.6
	0:100	Profit dairy farmer, million RMB/yr	-0.033	-0.036	-0.031	-0.031	-0.034	-0.031	-0.033
		Profit arable farmer, RMB/ha/yr	3281	3281	3281	3019	3542	1722	3930
		Corn saved, Mg DM/ha	8.1	7.8	8.3	8.1	8.1	8.1	8.1
10	WPCS:CSS	Land area needed, ha	15.6	15.7	15.0	15.6	15.6	15.6	15.6

	100:0	Profit dairy farmer, million RMB/yr	0.658	0.652	0.653	0.662	0.654	0.683	0.648
		Profit arable farmer, RMB/ha/yr	3281	3281	3281	3019	3542	1722	3930
		Corn saved, Mg DM/ha	0	0	0	0	0	0	0
	WPCS:CSS	Land area needed, ha	18.6	18.9	17.7	18.6	18.6	18.6	18.6
	75:25	Profit dairy farmer, million RMB/yr	0.670	0.664	0.666	0.675	0.665	0.692	0.661
		Profit arable farmer, RMB/ha/yr	3281	3281	3281	3019	3542	1722	3930
		Corn saved, Mg DM/ha	2.4	2.4	2.4	2.4	2.4	2.4	2.4
	WPCS:CSS5	Land area needed, ha	23.1	23.6	21.8	23.1	23.1	23.1	23.1
	0:50	Profit dairy farmer, million RMB/yr	0.688	0.680	0.684	0.694	0.682	0.706	0.681
		Profit arable farmer, RMB/ha/yr	3281	3281	3281	3019	3542	1722	3930
		Corn saved, Mg DM/ha	4.8	4.8	4.8	4.8	4.8	4.8	4.8
	WPCS:CSS2	Land area needed, ha	30.3	31.4	28.4	30.3	30.3	30.3	30.3
	5:75	Profit dairy farmer, million RMB/yr	0.718	0.709	0.713	0.726	0.710	0.730	0.713
		Profit arable farmer, RMB/ha/yr	3281	3281	3281	3019	3542	1722	3930
		Corn saved, Mg DM/ha	7.2	7.2	7.2	7.2	7.2	7.2	7.2
	WPCS:CSS	Land area needed, ha	32.1	32.1	30.8	32.1	32.1	32.1	32.1
	0:100	Profit dairy farmer, million RMB/yr	0.661	0.636	0.673	0.669	0.652	0.678	0.653
		Profit arable farmer, RMB/ha/yr	3281	3281	3281	3019	3542	1722	3930
		Corn saved, Mg DM/ha	7.6	7.2	7.8	7.6	7.6	7.6	7.6
20	WPCS:CSS	Land area needed, ha	21.5	21.6	20.6	21.5	21.5	21.5	21.5
	100:0	Profit dairy farmer, million RMB/yr	1.489	1.482	1.481	1.495	1.484	1.523	1.475
		Profit arable farmer, RMB/ha/yr	3281	3281	3281	3019	3542	1722	3930
		Corn saved, Mg DM/ha	0	0	0	0	0	0	0
	WPCS:CSS	Land area needed, ha	25.5	25.8	24.4	25.5	25.5	25.5	25.5
	75:25	Profit dairy farmer, million RMB/yr	1.507	1.498	1.499	1.513	1.500	1.536	1.494
		Profit arable farmer, RMB/ha/yr	3281	3281	3281	3019	3542	1722	3930
		Corn saved, Mg DM/ha	2.4	2.4	2.4	2.4	2.4	2.4	2.4
		Land area needed, ha	31.5	32.1	29.9	31.5	31.5	31.5	31.5

WPCS:CSS50:50	Profit dairy farmer, million RMB/yr	1.532	1.522	1.525	1.541	1.524	1.557	1.522
	Profit arable farmer, RMB/ha/yr	3281	3281	3281	3019	3542	1722	3930
	Corn saved, Mg DM/ha	4.8	4.8	4.8	4.8	4.8	4.8	4.8
WPCS:CSS25:75	Land area needed, ha	34.3	34.6	33.9	34.3	34.3	34.3	34.3
	Profit dairy farmer, million rmb/yr	1.487	1.469	1.503	1.496	1.478	1.515	1.475
	Profit arable farmer, RMB/ha/yr	3281	3281	3281	3019	3542	1722	3930
	Corn saved, Mg DM/ha	5.6	5.4	6.0	5.6	5.6	5.6	5.6
WPCS:CSS 0:100	Land area needed, ha	35.5	35.2	34.6	35.5	35.5	35.5	35.5
	Profit dairy farmer, million RMB/yr	1.411	1.383	1.427	1.421	1.402	1.447	1.396
	Profit arable farmer, RMB/ha/yr	3281	3281	3281	3019	3542	1722	3930
	Corn saved, Mg DM/ha	5.8	5.4	6.1	5.8	5.8	5.8	5.8
WPCS:CSS 100:0	Land area needed, ha	28.0	28.1	27.0	28.0	28.0	28.0	28.0
	Profit dairy farmer, million RMB/yr	2.311	2.302	2.299	2.318	2.303	2.354	2.293
	Profit arable farmer, RMB/ha/yr	3281	3281	3281	3019	3542	1722	3930
	Corn saved, Mg DM/ha	0	0	0	0	0	0	0
WPCS:CSS 75:25	Land area needed, ha	30.9	32.0	31.5	30.9	30.9	30.9	30.9
	Profit dairy farmer, million RMB/yr	2.290	2.297	2.314	2.298	2.282	2.335	2.271
	Profit arable farmer, RMB/ha/yr	3281	3281	3281	3019	3542	1722	3930
	Corn saved, Mg DM/ha	1.4	1.8	2.2	1.4	1.4	1.4	1.4
WPCS:CSS50:50	Land area needed, ha	32.1	32.8	32.4	32.1	32.1	32.1	32.1
	Profit dairy farmer, million RMB/yr	2.227	2.221	2.247	2.236	2.219	2.278	2.206
	Profit arable farmer, RMB/ha/yr	3281	3281	3281	3019	3542	1722	3930
	Corn saved, Mg DM/ha	1.8	1.9	2.5	1.8	1.8	1.8	1.8
WPCS:CSS25:75	Land area needed, ha	33.5	33.5	33.4	33.5	33.5	33.5	33.5
	Profit dairy farmer, million RMB/yr	2.159	2.141	2.176	2.168	2.151	2.217	2.135
	Profit arable farmer, RMB/ha/yr	3281	3281	3281	3019	3542	1722	3930
	Corn saved, Mg DM/ha	2.2	2.1	2.7	2.2	2.2	2.2	2.2
WPCS:CSS	Land area needed, ha	34.9	34.4	34.4	34.9	34.9	34.9	34.9

0:100	Profit dairy farmer, million RMB/yr	2.086	2.058	2.101	2.095	2.076	2.151	2.058
	Profit arable farmer, RMB/ha/yr	3281	3281	3281	3019	3542	1722	3930
	Corn saved, Mg DM/ha	2.7	2.2	3.0	2.7	2.7	2.7	2.7

DM, dry matter; WPCS, whole plant corn silage.

[a]Default situation was when the chemical composition of WPCS and CSS were the average of the four cultivars.

[b]The metabolizable energy value of CSS of Perley is greatest (9.46 MJ/kg DM) and of Rivaldinio is lowest (8.41 MJ/kg DM), predicted by NRC [20] at maintenance level.

[c]The greatest and lowest price of ground corn during 2015 was 2.13 and 2.47 RMB/kg fresh weight, respectively.
